# Noninvasive Assessment of Cardiopulmonary Hemodynamics Using Cardiovascular Magnetic Resonance Pulmonary Transit Time

**DOI:** 10.1155/2024/5691909

**Published:** 2024-10-28

**Authors:** Martin Segeroth, David Jean Winkel, Beat A. Kaufmann, Ivo Strebel, Shan Yang, Joshy Cyriac, Jakob Wasserthal, Michael Bach, Pedro Lopez-Ayala, Alexander Sauter, Christian Mueller, Jens Bremerich, Michael Zellweger, Philip Haaf

**Affiliations:** ^1^Department of Radiology and Nuclear Medicine, University Hospital Basel and University of Basel, Basel, Switzerland; ^2^Department of Cardiology, Cardiovascular Research Institute Basel, University Hospital Basel and University of Basel, Basel, Switzerland; ^3^Department of Research and Analysis, University Hospital Basel and University of Basel, Basel, Switzerland

**Keywords:** cardiac magnetic resonance imaging (CMR), diastolic dysfunction, echocardiography, heart failure, mitral valve regurgitation, pulmonary transit time (PTT)

## Abstract

**Introduction:** Pulmonary transit time (PTT) is the time it takes blood to pass from the right ventricle to the left ventricle via the pulmonary circulation, making it a potentially useful marker for heart failure. We assessed the association of PTT with diastolic dysfunction (DD) and mitral valve regurgitation (MVR).

**Methods:** We evaluated routine stress perfusion cardiovascular magnetic resonance (CMR) scans in 83 patients including assessment of PTT with simultaneously available echocardiographic assessment. Relevant DD and MVR were defined as exceeding Grade I (impaired relaxation and mild regurgitation). PTT was determined from CMR rest perfusion scans. Normalized PTT (nPTT), adjusted for heart rate, was calculated using Bazett's formula.

**Results:** Higher PTT and nPTT values were associated with higher grade DD and MVR. The diagnostic accuracy for the prediction of DD as quantified by the area under the ROC curve (AUC) was 0.73 (CI 0.61–0.85; *p* = 0.001) for PTT and 0.81 (CI 0.71–0.89; *p* < 0.001) for nPTT. For MVR, the diagnostic performance amounted to an AUC of 0.80 (CI 0.68–0.92; *p* < 0.001) for PTT and 0.78 (CI 0.65–0.90; *p* < 0.001) for nPTT. PTT values < 8 s rule out the presence of DD and MVR with a probability of 70% (negative predictive value 78%).

**Conclusion:** CMR-derived PTT is a readily obtainable hemodynamic parameter. It is elevated in patients with DD and moderate to severe MVR. Low PTT values make the presence of DD and MVR—as assessed by echocardiography—unlikely.

## 1. Introduction

Heart failure (HF) is a leading cause of death and disability [1]. Its prevalence is approximately 1%–2% of the adult population in developed countries and reaches more than 10% among people over 70 years [1–3]. Major uncertainties remain regarding the definition and phenotyping of HF with both reduced and preserved ejection fraction, contributing to unacceptable high rates of mortality and morbidity [1–3].

Cardiovascular magnetic resonance (CMR) is increasingly used to differentiate the heterogeneous aetiology of HF and to guide treatment [4]. CMR uniquely provides a comprehensive assessment of morphology, function, perfusion, viability, and tissue characterization all in a single examination [4]. Recent advances in CMR enable early detection, quantification, and the distinction between permanent replacement fibrosis and dynamic interstitial fibrosis in HF using parametric mapping methods [5].

HF is often associated with relevant diastolic dysfunction (DD) or mitral valve regurgitation (MVR). However, comprehensive assessment of DD and exact quantification of MVR are typically not included in routine CMR scans. Instead, their evaluation in clinical practice relies mainly on indirect parameters such as dilatation of left-sided chambers, deterioration of LV function, or subjective and error-prone visual qualitative assessment of MVR [6].

Pulmonary transit time (PTT) is defined as the time required for blood to pass from the right ventricle (RV) to the left ventricle (LV) via the pulmonary circulation, and it can be measured by administering an intravenous contrast agent and detecting the bolus in both ventricles. PTT is influenced by factors such as preload, blood volumes, global diastolic and systolic function, valvular dysfunction, and pulmonary microcirculation. It serves as a noninvasive method to quantify cardiopulmonary haemodynamics [7, 8]. PTT has been demonstrated to be altered in various diseases, including HF [9, 10], pulmonary hypertension [11, 12], chronic lung disease [13], and mitral stenosis [14], and to correlate with cardiac function [15].

**Figure 1:**
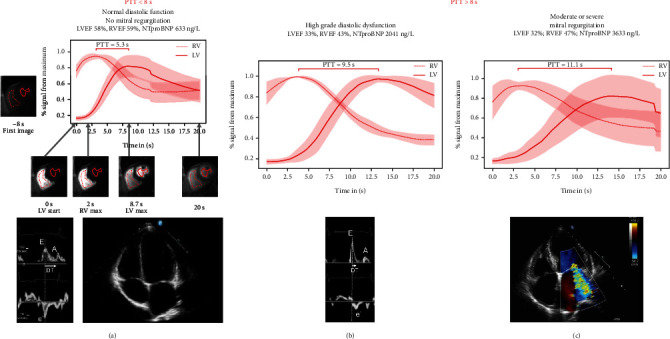
(a) Patients with a pulmonary transit time (PTT) below 8 s and normal diastolic function and no mitral regurgitation. Apart from the time signal curves (TSC) ROI placements for the right and left ventricular blood pool are displayed. At the bottom exemplary echocardiographic data (E/A wave, E/E' and a four-chamber view) is shown. (b) Patients with a PTT > 8 s and a high-grade diastolic dysfunction showing the TSCs and exemplary E/A wave and E/E'. (c) Patients with a PTT > 8 s and a moderate or severe mitral regurgitation showing the TSCs and exemplary four-chamber view with significant mitral valve regurgitation.

In this manuscript, we aimed to evaluate CMR-derived PTT—the time it takes blood to pass from the RV to the LV via the pulmonary circulation—as a novel tool to detect relevant DD and MVR (Figure 1).

## 2. Methods

### 2.1. Patients and Study Design

All patients referred for routine stress perfusion CMR to the University Hospital Basel (Switzerland) with available transthoracic echocardiography (TTE) within 1 month and digital ECGs within 1 day between January 2014 and August 2020 were enrolled for this retrospective study. Clinical data were retrieved from the electronic patients' records. Extracted data was transferred to a REDCap database (Research Electronic Data Capture; https://project-redcap.org; Vanderbilt University/IC 6.9.4). A final diagnosis was adjudicated to each patient by at least two board-certified cardiologists and radiologists based on the CMR perfusion scan and available medical records (including patient history, electrocardiogram, and results of laboratory testing and other imaging studies such as echocardiography or computed tomography and coronary angiography). The study was carried out according to the principles of the Declaration of Helsinki and approved by the local ethics committee, which waived the need for written informed consent (Req-2020-01001).

### 2.2. Echocardiographic Analysis and Assessment of DD

For all participants, a comprehensive TTE was performed either on an iE33 or EPIQ CVx ultrasound machine (Philips, Amsterdam, The Netherlands) equipped with X5-1 transducers and assessed by board-specified cardiologists. Left ventricular ejection fraction (LVEF), LV diastolic function, and valve function were assessed according to the American Society of Echocardiography guidelines [16, 17].

For this study, DD was defined as more than Grade I (impaired relaxation) DD. MVR was defined as more than Grade I (mild regurgitation).

### 2.3. CMR—Scan Protocol

CMR studies were performed on a 1.5 T or 3 T (MAGNETOM Avanto/Avanto fit, resp., Skyra, Siemens Healthineers, Erlangen, Germany). A standard clinical protocol, including cine–balanced steady-state free precession (bSSFP) imaging, adenosine stress, and rest perfusion followed by late gadolinium enhancement, was acquired for all studies with ECG-triggering. The cine sequences at 3 T had the following parameters: TE 1.47–1.61 ms; flip angle 48°–63°; pixel bandwidth 780; TR 40.32–44.16 ms; spacing between slices 7.2 mm. At 1.5 T, the parameters were as follows: TE 1.1–1.15 ms; flip angle 53°–74°; pixel bandwidth 930; TR 41.44–43.36; spacing between slices 11.04 mm. The myocardial perfusion imaging sequence was a single-shot saturation-recovery spoiled gradient echo. Basal, midventricular, and apical short-axis perfusion images were acquired at both stress and rest. Images were acquired during 60–75 heartbeats with ECG triggering. The temporal resolution of the perfusion scan varied with heart beats per minute of each patient and was 1.16 s (IQR: 0.88 –1.97 s) for this study. The perfusion sequences at 3T had the following parameters: TE 1.03 ms; flip angle 10°; pixel bandwidth 1000; TR 158–177 ms; spacing between slices 9.6 mm. At 1.5T the parameters were: TE 1.17 ms; flip angle 12°; pixel bandwidth 1000; TR 149–174 ms; spacing between slices 9.6 mm. A bolus of 0.1 mmol/kg gadoterate meglumine was administered.

### 2.4. CMR Volumetric Analysis

All CMR scans and biventricular cardiac volume parameters were clinically assessed by joint reporting of at least two board-certified physicians (cardiologist and radiologist) with a commercially available software (syngo.via VB40, Siemens Healthineers, Erlangen, Germany). Biventricular volumetry was performed as proposed in current publications [18]. For all volumetric analyses, papillary muscles were consistently included in the ventricular blood volume [18].

### 2.5. CMR PTT

PTT was calculated from rest perfusion images with motion correction (MoCo). For the analysis, Nora, a web-based framework for medical image analysis, was used [19] (Figure 1(a), Video S1). PTT was determined by placing regions of interest (ROIs) in the blood pools of LV and RV–sparing papillary muscles. The ROI of the LV and RV were determined automatically by a recently published deep learning–based biomedical image segmentation [20] using an nnU-Net [21]. PTT was determined from rest perfusion scans as the time in seconds between peak signal intensities of the time signal curves of the RV and LV, with the exclusion of the recirculation component. We chose to calculate PTT with the peak-to-peak method as it has been established in previous studies [22–24].

PTT was normalized using Bazett's formula as suggested in previous studies [25, 26] by dividing the PTT by the square root of the duration of the cardiac cycle (R-R interval). Patients' heart rates at rest were derived from the CMR scans.

### 2.6. Statistical Analysis

The time signal curves were reported as means +/− SD. Since no variable was normally distributed, all are reported as medians (interquartile range (IQR)). Normality was verified by a visual approach using frequency histograms and quantification using the Kolmogorov−Smirnov test. Comparison between groups was performed for continuous variables using a two-tailed unpaired Student's *t*-test or a rank-sum test depending on normality. Hypothesis testing was two-tailed. More than two groups were compared using the Kruskal−Wallis test. All *p* values < 0.05 were considered statistically significant. In constructing quartiles of nPTT for a baseline characteristics table, the dataset was first sorted in ascending order, and quartile values (Q1, Q2, and Q3) were determined based on specific percentiles (25th, 50th, and 75th) of the dataset distribution. For evaluating the association between PTT/nPTT and E/A and E/e' ratio and between PTT/nPTT and TR velocity, scatterplots with a line of best fit were constructed. Correlations were assessed using Pearson's correlation coefficient and Spearman's rank correlation. The diagnostic accuracies as well as sensitivity and specificity were assessed by receiver operating characteristics curves. Confidence intervals were estimated using bootstrapping [27, 28]. All statistical analyses were performed using Python 3.8.8.

## 3. Results

### 3.1. Baseline Characteristics

Baseline characteristics and relation to quartiles of nPTT are illustrated in Table 1. The adjudicated final diagnosis was coronary artery disease in 37% of all patients, dilated cardiomyopathy in 8% of patients, acute (peri)myocarditis in 8%, and Takotsubo syndrome in 6%; 18% of patients had a normal CMR scan (see Table 1 for details).

Patients with higher nPTT values had lower ventricular ejection fractions, higher left end-diastolic volumes, and a lower indexed stroke volume. Patients with higher nPTT values more often had dilated cardiomyopathy (Table 1).

### 3.2. PTT in Patients Without Relevant DD, Mitral Valvular Regurgitation, and Normal Biventricular Ejection Fraction

The median time between CMR scan and echocardiography was 6 days (IQR: 2−30 days). Among the 83 patients who underwent additional echocardiographic assessment, 28 patients had no relevant DD (grade ≤ 1) and both left and right CMR ventricular ejection fraction above 50% (LVEF median 62%, IQR 57%−66%; RVEF median 62%, IQR 57%−68%). Their PTT values amounted to 6.9 s (IQR 6.0−7.9 s) and nPTT values to 6.6 (IQR 5.8−7.2).

Thirty-two patients had no relevant MVR (grade ≤ 1) and both left and right CMR ventricular ejection fraction above 50% (LVEF median 62%, IQR 55%−66%; RVEF median 62%, IQR 57%−68%). Their PTT values amounted to 6.9 s (IQR 6.0−7.9 s) and nPTT values to 6.6 (IQR 6.0−7.9).

The 45 patients with no relevant DD (grade ≤ 1) and no relevant MVR (grade ≤ 1) had an LVEF about 54% (IQR 46%−63%), an RVEF about 57% (IQR 51%−64%), and an SVI about 43 mL/m^2^ (IQR 35−50 mL/m^2^). Their PTT values amounted to 7.0 s (IQR 6.0−8.5 s) and nPTT values of 6.7 (IQR 5.9−7.9).

### 3.3. PTT for Evaluation of DD and MVR as Assessed by Echocardiography

**Figure 2:**
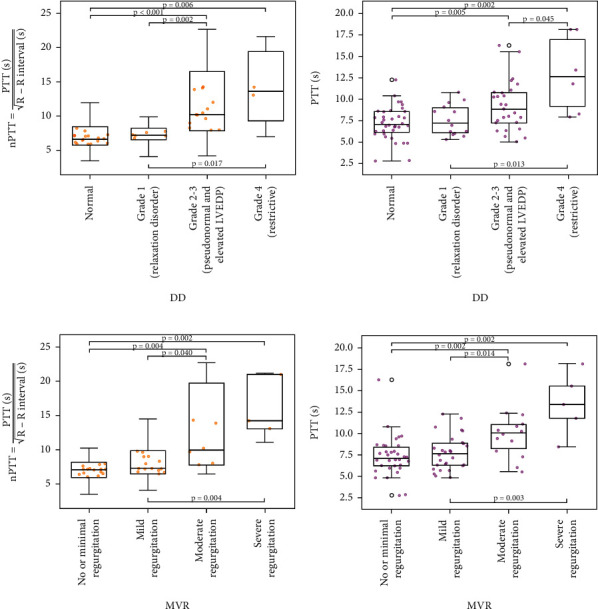
(a) Normalized pulmonary transit time (nPTT) according to groups of diastolic dysfunction (DD) and (b) pulmonary transit time (PTT) according to groups of DD, whereas nPTT and PTT increase with increasing DD; (c) nPTT according to groups of mitral valve regurgitation (MVR) and (d) PTT according to groups of MVR, whereas nPTT and PTT increase with increasing MVR.

Higher PTT and nPTT values were associated with increasing DD (Figures 2(a) and 2(b)) and increasing MVR (Figures 2(c) and 2(d)).

Thirty-six patients had a normal DD, 14 demonstrated a relaxation disorder, 27 had pseudonormal diastolic function or showed an elevated left ventricular end-diastolic pressure (LVEDP), and 6 were rated as restrictive.

Echocardiographic parameters of DD such as E/A ratio and E/e' ratio and tricuspid regurgitation (TR) velocity correlated with CMR PTT and nPTT values, but not with cardiac index (Figures S1–S4).

MVR was absent or minimal in 34 patients, mild in 28 patients, moderate in 14, and severe in 5.

### 3.4. Diagnostic Accuracy of CMR PTT for Assessment of DD as Assessed by Echocardiography

**Figure 3:**
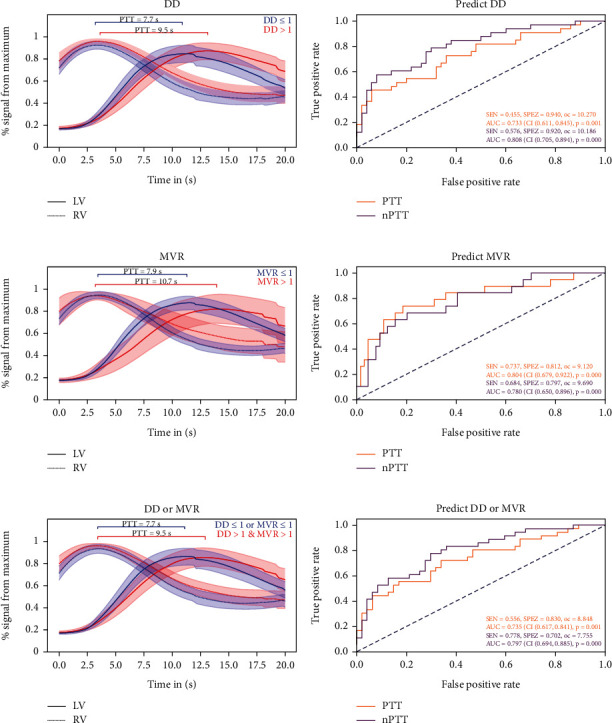
(a) Lower values of pulmonary transit time (PTT) of patients without relevant diastolic dysfunction (DD) (*n* = 33) compared to patients with DD (*n* = 50). (b) Diagnostic performance of PTT and normalized pulmonary transit time (nPTT) in receiver operating characteristic (ROC) curve analysis for the detection of DD. (c) Lower values of pulmonary transit time of patients without relevant MVR (*n* = 19) compared to patients with mitral valve regurgitation (MVR) (*n* = 64). (d) Diagnostic performance of PTT and nPTT in ROC curve analysis for the detection of MVR. (e) Lower values of pulmonary transit time of patients without relevant DD or MVR (*n* = 36) compared to patients with DD or MVR (*n* = 47). (f) Diagnostic performance of PTT and nPTT in ROC curve analysis for the detection of DD or MVR.

The diagnostic accuracy of PTT and nPTT for DD as quantified by the area under the ROC curve (AUC) was 0.73 (CI 0.61–0.85; *p* = 0.001) for PTT and 0.81 (CI 0.71–0.89; *p* < 0.001) for nPTT (Figures 3(a) and 3(b)). A specificity of 70% for DD was observed for a PTT < 8.8 s (negative predictive value (NPV) 77%) and an nPTT < 7.8 (NPV 88%). A sensitivity of 70% was observed for a PTT > 7.9 s (NPV 85%) and an nPTT > 7.9 (NPV 86%).

### 3.5. Diagnostic Accuracy of CMR PTT for Assessment of MVR as Assessed by Echocardiography

For MVR, the diagnostic performance of PTT and nPTT amounted to an AUC of 0.80 (CI 0.68–0.92; *p* < 0.001) for PTT and 0.78 (CI 0.65–0.90; *p* < 0.001) for nPTT (Figures 3(c) and 3(d)). A specificity of 70% for MVR was observed for a PTT < 9.1 s (NPV 93%) and an nPTT < 9.7 (NPV 91%). A sensitivity of 70% was observed for a PTT > 9.1 s (NPV 93%) and an nPTT > 8.0 (NPV 93%).

### 3.6. Diagnostic Accuracy of CMR PTT for Assessment of DD OR MVR as Assessed by Echocardiography

For the presence of both DD or MVR, the diagnostic performance of PTT and nPTT amounted to an AUC of 0.74 (CI 0.62 to 0.84; *p* = 0.001) for PTT and 0.80 (CI 0.69–0.89; *p* < 0.001) for nPTT (Figures 3(e) and 3(f)). A specificity of 70% was observed for a PTT < 8.0 s (NPV 78%) and an nPTT < 7.8 (NPV 85%). A sensitivity of 70% was observed for a PTT > 7.9 s (NPV 82%) and an nPTT > 7.9 (NPV 82%).

**Figure 4:**
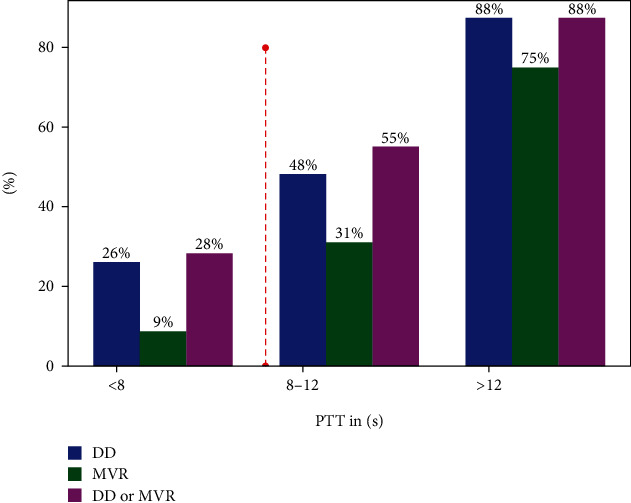
The percentage of patients with a diastolic dysfunction (DD) more than Grade 1 (impaired relaxation) increases with increasing PTT. Also, the percentage of patients with mitral valve regurgitation (MVR) more than mild regurgitation increases with increasing PTT. In the group of patients with a PTT < 8 s (*n* = 46), 13 (28%) had a DD or MVR in the group with a PTT between 8 and 12 s (*n* = 29), and 16 (55%) had a DD or MVR, and in the group with a PTT above 12 s (*n* = 8), 7 (88%) had a DD or MVR.

Using a cut-off value of PTT < 8 s resulted in a correct rule-out of DD and MVR in more than 70% of patients. 62% of patients with PTT > 8 s had either DD or MVR (Figure 4). In the group of patients with an PTT < 8 s (*n* = 46), only 13 (28%) had either DD or MVR.

## 4. Discussion

In this retrospective study of patients referred for routine stress perfusion CMR and echocardiographic assessment, we aimed to scrutinize PTT as an easily obtainable hemodynamic parameter to assess the likelihood of significant DD and MVR. We report four major findings.

First, patients with biventricular ejection fraction > 50% and absence of relevant DD or MVR presented with the lowest PTT values of 6–8 s. These values are similar to asymptomatic controls of other studies and might represent the normal range of PTT [29]. Second, the diagnostic accuracy of PTT and nPTT for detecting both relevant DD and MVR was moderate to high. Third, TTE parameters of DD such as E/A ratio and E/e' ratio and TR velocity also correlated positively with PTT and nPTT values. Fourth, using a cut-off value of PTT < 8 s resulted in a correct rule out of DD or MVR in more than 70% of patients.

Initially, PTT was derived invasively by right and left heart catheterization [14]. PTT can also be derived from computed tomography [15] and contrast–enhanced echocardiography [8, 30]. More recently, noninvasive methods to derive PTT from CMR [22, 29] have been deployed. The acquisition of rest first-pass perfusion images during the application of a Gadolinium–based contrast agent to acquire the PTT will not lead to a relevant prolongation of the CMR scan, allowing acquisition of PTT in clinical routine. Furthermore, it was demonstrated that PTT and nPTT are easily and automatically obtainable with a very low inter-rater variability [30, 31].

To the best of our knowledge, this is the first CMR study to assess and compare the diagnostic accuracy of PTT derived from CMR for the absence of DD and MVR as assessed by echocardiography.

Patients referred for stress perfusion CMR are often outpatients with unclear dyspnoea and without recent echocardiographic assessments. CMR is effective in identifying patients with coronary artery disease and is increasingly used for multi-parametric tissue characterization in patients with unclear HF. HF is frequently accompanied by relevant DD or MVR contributing or being the leading cause of patients' dyspnoea. Comprehensive assessments of DD and exact quantification of MVR are both not part of routine CMR scans. In clinical practice, their assessment relies mainly on indirect parameters such as dilatation of left-sided chambers or worsening LV function. Additionally, visual qualitative assessment of MVR is very susceptible to changes in cine pulse sequences and, therefore, is unreliable and should be used only cautiously [6]. Routine quantification of PTT could lead to a more comprehensive assessment of haemodynamic of patients with HF. A low PTT value (< 8 s) allowed to rule out a relevant DD or MVR in more than 70% of patients.

In a TTE pilot study, it was demonstrated that parameters of DD such as E/e' ratio and TR velocity correlated positively with PTT [30]. The authors observed that PTT may differentiate between stages of diastolic function. We confirm these findings and further demonstrate that PTT measured by CMR perfusion scan can differentiate between stages of DD as assessed by TTE.

The reason why markers of DD correlated with PTT but not cardiac output remains speculative: A possible explanation might be that an increase in LVEDP is a compensatory mechanism to maintain cardiac output. Conversely, PTT as a global but less specific marker of impaired haemodynamics might be susceptible to increasing values of LVEDP.

While quantification of MVR can be reached reliably and highly accurately using standard CMR methods (LV stroke volume minus aortic phase–contrast forward volume), flow measurements are not part of routine clinical HF scan protocols [6]. Animal studies demonstrated that PTT is prolonged in dogs with MVR as measured via gamma camera, even more so in dogs with decompensated MVR [32, 33]. We confirm and corroborate these findings by increasing PTT values with increasing grades of MVR in our study.

## 5. Limitations

First, as a retrospective study, we cannot quantify exactly the clinical benefit of PTT for the exclusion of relevant DD and MVR. Second, the sample size is limited since the majority of patients referred for CMR perfusion scans are outpatients without simultaneously available echocardiography. This may have led to a selection bias since only certain patients will get a simultaneous in-house echocardiographic assessment. Third, we only assessed patients with dyspnoea and/or suspected coronary artery disease referred for CMR stress perfusion. Fourth, possible saturation effects in rest perfusion images modifying the PTT were minimized by selecting the center of the ventricle as ROI by the automatic algorithm and by averaging over all voxels in the ROI for every specific time step.

## 6. Conclusion

PTT is a noninvasive marker of cardiopulmonary haemodynamics that can be easily obtained from routine CMR perfusion scans. It may be used as a single metric to make the presence of relevant DD and MVR unlikely. With a PTT < 8 s, we obtained a NPV of 80% for both pathologies.

## Figures and Tables

**Table 1:** Baseline characteristics of all patients and relating to pulmonary transition time (nPTT) quartiles.

	**All patients (** **n** = 83**)**	**Quartile 1 nPTT (< 6.5) (** **n** = 21**)**	**Quartile 2 nPTT (6.5–7.8) (** **n** = 21**)**	**Quartile 3 nPTT (7.8–10.2) (** **n** = 20**)**	**Quartile 4 nPTT (> 10.2) (** **n** = 21**)**	**p** ** value**
Demographics						
Age (years)	69 (53.5–78)	63 (47–74)	70 (61–80)	72 (61–81)	68 (53–78)	0.19
Sex (male) %	63%	52%	67%	65%	67%	0.93
Body surface area (m^2^)	1.8 (1.7–2.1)	1.9 (1.7–2.1)	1.8 (1.7–1.9)	1.9 (1.7–2.0)	1.8 (1.7–2.1)	0.73
Body mass index (kg/m^2^)	24.5 (22.5–28.1)	23.8 (22.2–28.4)	23.9 (22.8–25.3)	25.5 (22.3–27.9)	25.9 (23.7–29.7)	0.78
Diabetes mellitus (%)	35%	33%	31%	36%	38%	0.89
Hypercholesterolemia (%)	47%	67%	42%	55%	31%	0.80
Hypertension (%)	74%	83%	69%	83%	63%	0.99
History of myocardial infarction (%)	71%	67%	88%	64%	70%	0.99
CMR volumetric parameters						
LVEF (%)	49 (33–61)	59 (53–66)	47 (39–63)	50 (37–56)	28 (21–37)	<0.0001
RVEF (%)	55 (43–64)	62 (53–68)	55 (49–62)	58 (47–65)	41 (29–53)	0.002
EDVI LV (mL/m^2^)	89 (76–113)	79 (71–88)	92 (79–104)	85 (72–109)	129 (91–155)	0.002
EDVI RV (mL/m^2^)	76 (66–92)	75 (65–82)	80 (69–94)	70 (58–86)	82 (71–97)	0.14
Myocardial mass indexed (g/m^2^)	69 (61–87)	61 (54–72)	76 (61–87)	69 (66–83)	72 (65–108)	0.056
Stroke volume indexed (mL/m^2^)	42 (33–48)	46 (40–52)	45 (38–50)	40 (33–46)	32 (30–38)	0.004
Cardiac output (L/min)	3.3 (2.5–5.2)	3.1 (2.7–5.0)	3.2 (2.5–5.6)	3.8 (2.4–5.2)	3.6 (2.4–4.7)	0.99
Cardiac index (L/min × m^2^)	1.8 (1.4–2.8)	1.6 (1.4–2.8)	1.9 (1.4–3.0)	2.1 (1.3–2.6)	1.7 (1.3–2.5)	0.87
CMR late gadolinium enhancement (LGE)						
Myocardial infarction (ischaemic LGE pattern)	24%	19%	33%	25%	19%	0.75
Nonischaemic Fibrosis (nonischaemic LGE pattern)	24%	19%	19%	10%	48%	0.066
Final adjudicated diagnosis						
Coronary artery disease (%)	37%	33%	43%	40%	33%	0.95
Dilated cardiomyopathy (%)	8%	0%	5%	5%	24%	0.03
Acute (peri-) myocarditis (%)	8%	14%	5%	10%	5%	0.67
Takotsubo syndrome (%)	6%	5%	0%	15%	5%	0.28
Hypertrophic cardiomyopathy (%)	4%	5%	5%	0%	5%	0.80
Normal CMR scan (%)	18%	38%	5%	20%	10%	0.053
Unclear diagnosis (%)	11%	5%	24%	10%	5%	0.19
Other (%)	5%	0%	10%	0%	10%	0.26

*Note:* Values are displayed as median (interquartile range) or %. Information about comorbidities was available for 58% of patients.

Abbreviations: CMR = cardiovascular magnetic resonance, EDVI = end diastolic volume indexed, LV = left ventricle, LVEF = left ventricular ejection fraction, PCI = percutaneous coronary intervention, RV = right ventricle, RVEF = right ventricular ejection fraction.

## Data Availability

Data is available on request to the authors.

## Nomenclature

CMR: cardiac magnetic resonance imaging
DD: diastolic dysfunction
HF: heart failure
IQR: interquartile range
LV: left ventricle
MVR: mitral valve regurgitation
nPTT: normalized pulmonary transit time
PTT: Pulmonary transit time
ROI: region of interest
RV: right ventricle

## Supporting Information

Supporting Information 1 Figure S1: (a) correlation between normalized pulmonary transition time (nPTT) and E/A ratio; (b) nPTT according to E/A ratio; (c) correlation between pulmonary transit time (PTT) and E/A ratio; (d) PTT according to E/A ratio. Figure S2: (a) correlation between normalized pulmonary transit time (nPTT) and E/e' ratio; (b) nPTT according to E/e' ratio; (c) correlation between pulmonary transit time (PTT) and E/e' ratio; (d) PTT according to E/e' ratio. Figure S3: (a) correlation between normalized pulmonary transit time (nPTT) and TR velocity; (b) nPTT according to TR velocity; (c) correlation between pulmonary transit time (PTT) and TR velocity; (d) PTT according to TR velocity. Figure S4: (a) correlation between cardiac index and E/A ratio; (b) correlation between cardiac index and E/e' ratio; (c) correlation between cardiac index and TR velocity.

Supporting Information 2 Video S1: signal change in the ventricles of a patient with normal (PTT < 8 s) and prolonged PTT (PTT > 8 s), demonstrating the faster signal change with a PTT below 8 s.
