# Supplementary material for: Noninvasive Assessment of Cardiopulmonary Hemodynamics Using Cardiovascular Magnetic Resonance Pulmonary Transit Time
Source: Int J Biomed Imaging. 2024 Oct 28;2024:5691909. doi: 10.1155/2024/5691909 (PMC11535428; doi:10.1155/2024/5691909)
Supplement: Supporting Information 1 — Figure S1: (a) correlation between normalized pulmonary transition time (nPTT) and E/A ratio; (b) nPTT according to E/A ratio; (c) correlation between pulmonary transit time (PTT) and E/A ratio; (d) PTT according to E/A ratio. Figure S2: (a) correlation between normalized pulmonary transit time (nPTT) and E/e' ratio; (b) nPTT according to E/e' ratio; (c) correlation between pulmonary transit time (PTT) and E/e' ratio; (d) PTT according to E/e' ratio. Figure S3: (a) correlation between normalized pulmonary transit time (nPTT) and TR velocity; (b) nPTT according to TR velocity; (c) correlation between pulmonary transit time (PTT) and TR velocity; (d) PTT according to TR velocity. Figure S4: (a) correlation between cardiac index and E/A ratio; (b) correlation between cardiac index and E/e' ratio; (c) correlation between cardiac index and TR velocity. [file 5691909.f1.docx]

**Supplement**

**
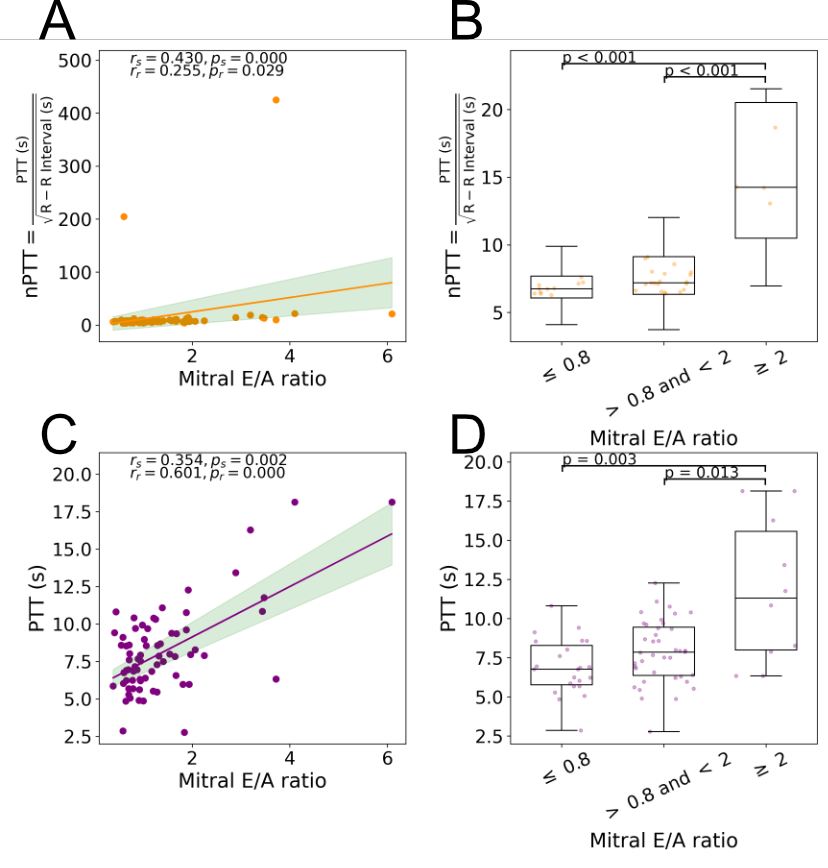
**

**Supplement Figure 1S:** A) Correlation between normalized pulmonary transition time (nPTT) and E/A ratio; B) nPTT according E/A ratio; C) Correlation between pulmonary transit time (PTT) and E/A ratio; D) PTT according to E/A ratio.

**
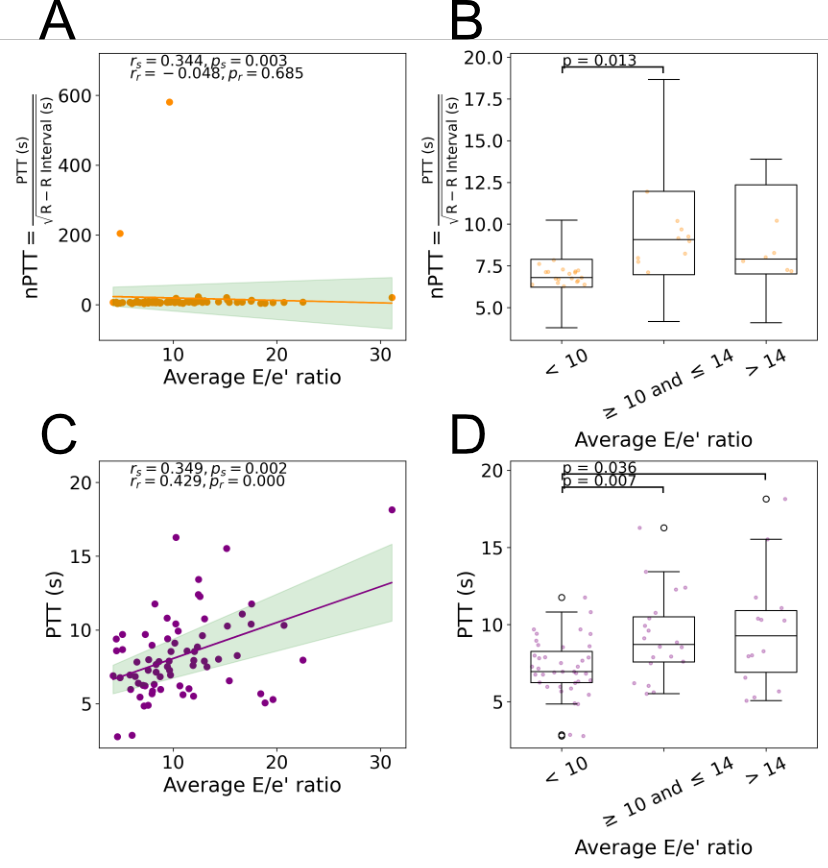
**

**Supplement Figure 2S:** A) Correlation between normalized pulmonary transit time (nPTT) and E/e’ ratio; B) nPTT according to E/e’ ratio; C) Correlation between pulmonary transit time (PTT) and E/e’ ratio; D) PTT according to E/e’ ratio.

**
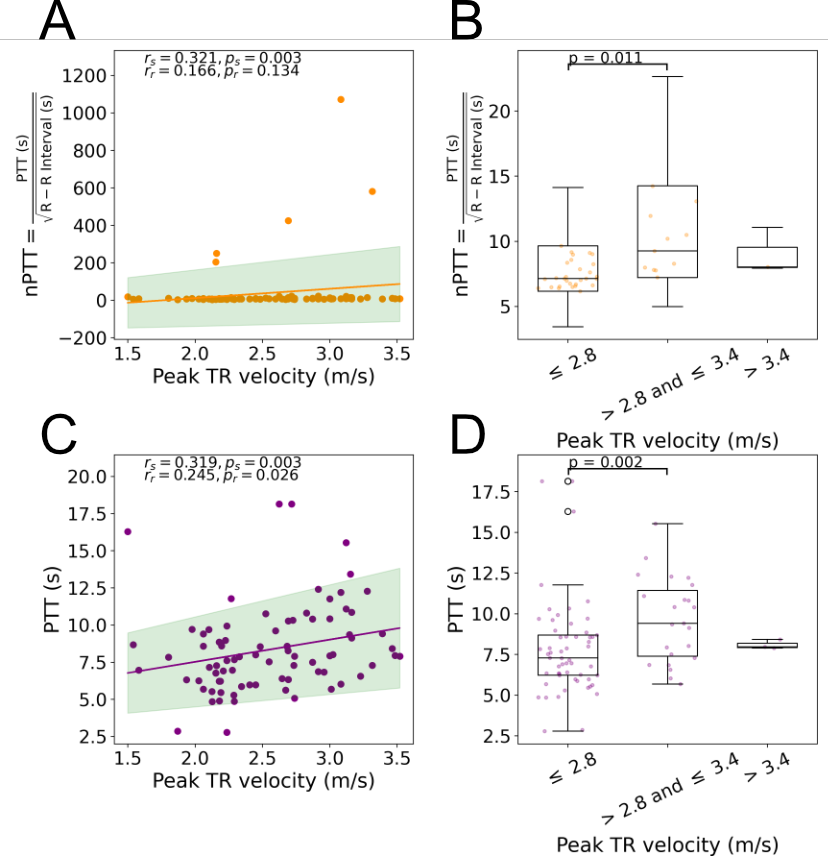
**

**Supplement Figure 3S:** A) Correlation between normalized pulmonary transit time (nPTT) and TR velocity; B) nPTT according to TR velocity; C) Correlation between pulmonary transit time (PTT) and TR velocity; D) PTT according to TR velocity.


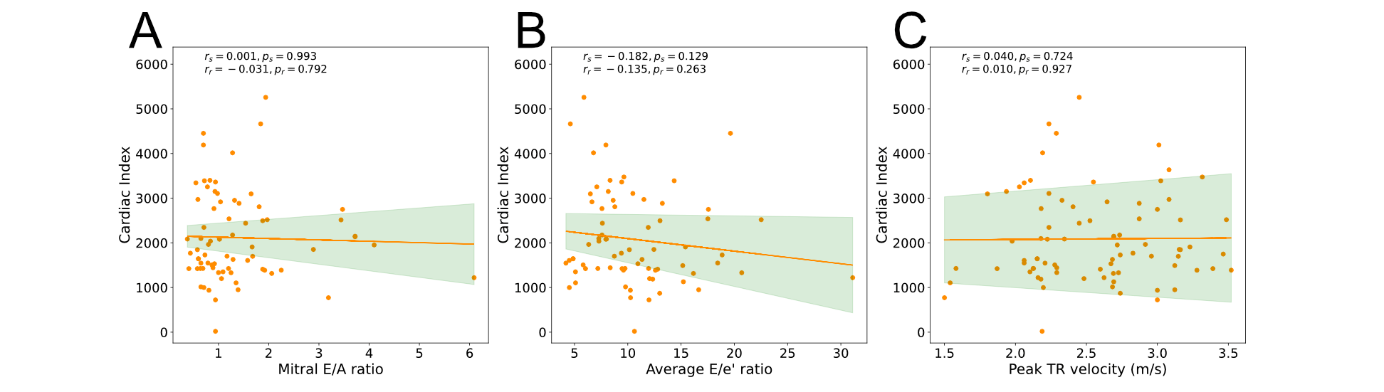


**Supplement Figure 4S:** A) Correlation between Cardiac index and E/A ratio; B) Correlation between Cardiac index and E/e’ ratio; C) Correlation between Cardiac index and TR velocity.
